# Trained immunity as a systemic bridge: the liver-gut-immune-oral axis in the comorbidity of chronic liver disease and periodontitis

**DOI:** 10.3389/fimmu.2026.1855561

**Published:** 2026-06-04

**Authors:** Xiang Li, Guodong Lv, Daolang Yuan, Jun Hu, Wenrong Lou, Yulin Tu, Jing Zhou, Rong Qin

**Affiliations:** 1 Kunming Medical University, Kunming, Yunnan, China; 2 Calmette Hospital Affiliated to Kunming Medical University, Kunming, Yunnan, China; 3 Department of Otorhinolaryngology, Yan’an Hospital Affiliated to Kunming Medical University, Kunming, Yunnan, China; 4 Key Laboratory of Engineering and Translational Application of Cell Derivatives of Yunnan Province, Kunming, Yunnan, China; 5 Department of Stomatology, Yan’an Hospital Affiliated to Kunming Medical University, Kunming, Yunnan, China; 6 Department of Gastroenterology, Yan’an Hospital Affiliated to Kunming Medical University, Kunming, Yunnan, China

**Keywords:** Chronic liver disease, comorbidity, liver-gut-immune-oral axis, periodontitis, trained immunity

## Abstract

Trained immunity (TI) reveals that innate immune cells acquire long-term functional memory through metabolic and epigenetic reprogramming. This review examines TI in chronic liver diseases and periodontitis, proposing the “Liver-Gut-Immune-Oral Axis” as a framework where TI bridges these comorbidities. The forward pathway, currently inferred from mechanistic and associative studies, proposes that liver-gut dysfunction induces bone marrow training, generating hyper-reactive monocytes that amplify periodontal inflammation. The reverse pathway, similarly conceptual, proposes that periodontal pathogens reprogram hematopoietic progenitors, accelerating liver disease progression. Both converge on shared metabolic-epigenetic reprogramming circuits. We emphasize that this axis represents a conceptual framework synthesized from current mechanistic and associative evidence; its validity as an integrated, causally-linked biological system awaits direct experimental validation. Targeting TI with metabolic modulators, epigenetic drugs, or periodontal interventions offers strategies to disrupt this cycle and advance precision medicine for inflammatory comorbidities.

## 1 Introduction

TI has transformed the classical view that immunological memory is exclusive to adaptive immunity, demonstrating that innate immune cells establish long-term functional enhancement through metabolic and epigenetic remodeling (1, 2). This memory enables rapid, vigorous inflammatory responses upon re-stimulation, but when dysregulated, perpetuates chronic tissue damage (3, 4). Chronic liver diseases and periodontitis exemplify this pathological interplay. Liver diseases affect over 800 million people globally, driven by innate immune dysregulation (5). Periodontitis, affecting 50% of adults, is the leading cause of tooth loss (6). Clinically, these conditions coexist and mutually exacerbate, yet mechanistic links remain elusive (7, 8).

TI may bridge this gap. In liver disease, gut-derived PAMPs induce systemic trained states in myeloid cells through metabolic and epigenetic remodeling (3). These trained cells circulate and amplify periodontal inflammation upon secondary microbial challenge. Conversely, periodontal infection trains immune cells that traffic to the liver, accelerating hepatic inflammation through synergistic interactions with Kupffer cells and endotoxemia, establishing a self-perpetuating cycle (9). Importantly, TI represents one component of a broader immunopathological network; adaptive immunity, direct bacterial translocation, and metabolic dysregulation also contribute substantially to this bidirectional relationship, and the relative contribution of TI likely varies by disease stage and etiology (10–12).

This review synthesizes TI’s role in these diseases. Based on converging lines of mechanistic and associative evidence, we hypothesize that TI acts as a systemic bridge connecting these organs via a ‘Liver-Gut-Immune-Oral Axis.’ We explicitly position this as a testable framework: it integrates currently disparate findings into a coherent model, delineates TI’s molecular foundations and disease-specific manifestations, and maps hypothesized forward and reverse pathways of organ crosstalk. We acknowledge that these pathways are currently inferred from separate bodies of literature rather than single multimodal cohort studies, and that adaptive immunity, direct bacterial translocation, and metabolic dysregulation also contribute substantially to this bidirectional relationship. The relative contribution of TI, versus these other mechanisms, is likely to vary by disease stage and etiology and remains to be determined empirically.

## 2 Basic concepts of TI

### 2.1 The concept of TI

TI delineates the process through which innate immune cells, including monocytes and macrophages, experience metabolic reprogramming and enduring epigenetic modifications after their initial exposure to stimuli such as PAMPs, damage-associated molecular patterns (DAMPs), or specific cytokines. This process culminates in the formation of a long-term functional memory (13). This concept challenges the conventional notion that immunological memory is exclusively a characteristic of the adaptive immune system, revealing that innate immunity also possesses memory-like properties. Upon re-exposure to identical or distinct stimuli weeks or months later, these trained cells can initiate a more rapid and robust inflammatory response, thereby enhancing the host’s defense against subsequent infections. This stimulus-induced innate immune memory functions independently of the antigen-specific recognition typical of T and B lymphocytes, arising from intrinsic functional remodeling of innate immune cells. This perspective offers a novel understanding of the mechanisms underlying host immune defense (14, 15).

### 2.2 Metabolic reprogramming – the core driver of TI

TI is characterized by metabolic reprogramming, manifested as a transition in energy metabolism from oxidative phosphorylation to aerobic glycolysis, referred to as the Warburg effect. This metabolic pathway not only facilitates rapid energy production but also generates metabolites such as lactate and succinate, which can directly promote the expression of inflammatory genes. In inflammatory contexts, succinate has been shown to exert pro-inflammatory effects; for instance, dietary succinate was found to modulate colitis severity via the IL-4Rα/HIF-1α axis in a murine model (16). Within the specific framework of trained immunity, succinate accumulation is recognized as a key metabolic feature that stabilizes HIF-1α and supports the trained phenotype356 (3, 17), though whether it acts through IL-4Rα in this context requires confirmation. Within the tumor microenvironment, HIF-1α further amplifies glycolysis by regulating mitophagy (18). The central signaling pathways mediating this metabolic switch are detailed in Section 1.4 (19, 20). These findings suggest that metabolic alterations are not merely responses to energy demands but actively contribute to the establishment of TI at the signaling level.

### 2.3 Epigenetic remodeling – the key to maintaining the memory state

Concomitant with metabolic alterations, TI necessitates stable epigenetic remodeling to maintain long-term memory. Activating histone modifications, such as H3K4me3 and H3K27ac, are established in critical regions of inflammatory genes, including TNF-α and IL-6, thereby priming these genes for rapid activation upon subsequent stimulation (21). These modifications are directly influenced by metabolites, such as acetyl-CoA and α-ketoglutarate, which modulate the activity of epigenetic enzymes. Infection models indicate that the PI3K/AKT pathway can affect deacetylases like HDAC7, resulting in epigenetic features akin to TI in gingival cells (22). Furthermore, inhibiting histone methyltransferases such as MLL1 can significantly diminish trained immunity in experimental models (23), and genome-wide chromatin accessibility mapping has revealed disease-specific enhancer landscapes in trained monocytes (24). These findings underscore the pivotal role of epigenetic mechanisms.”

### 2.4 mTOR–HIF-1α – the central hub of TI

The mTOR–HIF-1α signaling axis integrates metabolic states, environmental stimuli, and gene regulation, functioning as the central hub of TI. Activation of mTOR stabilizes HIF-1α, thereby promoting glycolysis, inflammatory gene expression, and inflammasome activation; for instance, TREM-1 enhances NLRP3 activity through this pathway (25). In models utilizing β-glucan, inhibition of mTOR significantly reduces the metabolic phenotype associated with TI (26). Notably, HIF-1α also provides feedback regulation to mTOR, establishing a complex bidirectional circuit (27). mTOR inhibitors, such as rapamycin, can obstruct TI-like responses, suggesting potential avenues for future therapeutic interventions (20).

### 2.5 Pathophysiological significance of TI

TI enhances host defense against infections; however, it may also exacerbate inflammatory responses in chronic or systemic diseases. For example, in a model of liver disease and periodontitis comorbidity, liver-derived PAMPs disseminate systemically through the “liver-bone marrow axis,” inducing a uniform pro-inflammatory trained state in distal myeloid cells and thereby sustaining inflammation in tissues such as the periodontium (28). Similar phenomena have been documented in atherosclerosis and systemic lupus erythematosus, indicating that TI may serve as a fundamental mechanism driving multi-organ inflammatory synergy (29, 30).

### 2.6 Trained immunity and immune tolerance: a critical distinction

While TI offers a compelling framework for understanding innate immune memory, its application as a unifying concept requires critical scrutiny of its conceptual boundaries and relationship with immune tolerance (31, 32).

First, TI is an umbrella term covering divergent phenomena. Protective TI induced by β-glucan (dectin-1/RAF1 pathway) differs fundamentally from the maladaptive, pro-inflammatory memory driven by persistent LPS or periodontal PAMPs (TLR4/MyD88/NF-κB pathway). Conflating these risks obscuring therapeutic implications (33, 34).

Second, TI and immune tolerance (endotoxin tolerance) are not binary opposites but represent a dynamic spectrum (35). In chronic liver disease, prolonged low-grade endotoxemia may induce tolerance in Kupffer cells while simultaneously training bone marrow progenitors (36). Patients with advanced cirrhosis can exhibit a mixed phenotype—hyper-responsiveness for IL-1β (TI-like) alongside suppressed TNF-α and low HLA-DR (tolerance-like) (36). Therapies targeting TI without considering this coexistence risk unintentionally reinforcing tolerance and impairing host defense (36).

Third, current biomarkers lack specificity. H3K4me3 enrichment, often cited as a TI hallmark, also occurs in transient activation and varies in persistence across cell types. Without standardized thresholds, over-interpretation of epigenetic data is a real risk (37).

In summary, future studies must dissect the training-tolerance spectrum and validate cell-type-specific signatures rather than applying TI uncritically.

## 3 TI in liver disease

The liver, essential for immune tolerance, can shift from immune silence to pro-inflammatory memory under persistent stimuli like metabolites and inflammatory signals, exacerbating tissue inflammation (38). This process is crucial in chronic viral hepatitis, metabolic dysfunction-associated fatty liver disease, cirrhosis, and hepatocellular carcinoma, connecting local liver pathology with systemic inflammatory responses (39). TI plays a significant role in the pathogenesis and progression of liver diseases.

### 3.1 Chronic hepatitis and TI

Chronic hepatitis, primarily including chronic hepatitis B(CHB)and C, is an inflammatory liver disease caused by persistent infection with the respective viruses. Globally, over 800,000 people die annually from HBV infection-related liver failure and hepatocellular carcinoma (40). In this pathological context, TI, a mechanism intended to be protective, can become dysregulated, transforming from a defense force into a driver of tissue damage (41).

Patients with chronic hepatitis frequently exhibit persistent low-grade systemic inflammation. In addition to adaptive immune responses, long-term alterations in the innate immune system play a critical role in this phenomenon (42). The continuous presence of viral antigens may aberrantly “train” the innate immune system. Patients with CHB frequently exhibit persistent low-grade systemic inflammation (43), and the continuous presence of viral antigens may aberrantly prime the innate immune system. Some studies suggest that monocytes from CHB patients can exhibit heightened cytokine production upon stimulation (43), although direct evidence for classical trained immunity hallmarks—such as enhanced secondary responses to unrelated stimuli with associated H3K4me3 enrichment—in CHB remains limited. Whether this represents durable innate immune memory as opposed to persistent antigen-driven activation remains to be formally demonstrated. The persistent activation of TLR signaling by viral antigens may provide a basis fo innate immune remodeling (43); however, direct demonstration of TI-consistent epigenetic and metabolic signatures (see Section 1) specifically in CHB patient monocytes remains to be established. These findings suggest that the aberrant establishment of TI is a key factor in sustaining the inflammatory microenvironment in chronic hepatitis. Theoretically, this inappropriately amplified innate immune response could directly or indirectly exacerbate hepatocyte damage and fibrotic processes through the prolonged release of inflammatory mediators. Furthermore, TI may influence the function of intrahepatic Kupffer cells, thereby disrupting the liver’s regenerative and repair capacity, which ultimately impacts disease prognosis (44). Nevertheless, conflicting findings exist (45). While some studies show TI-like hyper-responsiveness in CHB monocytes, others report immune exhaustion or tolerance, particularly in patients with high viral loads (46). The determinants tipping the balance toward TI versus tolerance — antigen concentration, co-stimulatory signals, host genetics — remain poorly characterized (45).

The abnormal activation of TI impacts the inflammatory response in chronic hepatitis and can significantly influence the effectiveness and outcome of antiviral treatment. Importantly, despite successful viral suppression with nucleoside analogues, monocyte functional defects-–including impaired phagocytosis and altered cytokine production-–persist in CHB patients (45, 47), raising the possibility that maladaptive innate immune memory may remain incompletely reversed and contribute to disease progression after treatment discontinuation (48, 49).

Whether this represents durable trained immunity (with glycolytic switching and H3K4me3 enrichment), immune tolerance (with suppressed HLA-DR and TNF-α), or a mixed phenotype as described in cirrhosis (Section 2.3) remains unresolved (33, 45). This ambiguity has direct therapeutic implications: epigenetic modulators may be indicated for true TI, whereas tolerance reversal would require different strategies. Furthermore, this persistent inflammatory milieu could modify the liver’s immune environment, impeding full viral eradication and creating challenges for achieving clinical remission.

### 3.2 Metabolic dysfunction-associated steatotic liver disease (MASLD, previously termed non-alcoholic fatty liver disease, NAFLD) and TI

The most widespread chronic liver disease worldwide is MASLD, which spans from simple liver fat buildup to non-alcoholic steatohepatitis (NASH) with associated inflammation and fibrosis (50). While lipid metabolism disorders and insulin resistance are recognized core drivers of MASLD, recent research indicates that functional remodeling of the innate immune system with “memory” characteristics, mediated by TI, may also participate in the formation and maintenance of the hepatic lipotoxic microenvironment (41). However, TI operates within a complex network of disease drivers—including insulin resistance, adipokine dysregulation, lipotoxicity, and gut microbiome alterations—and its relative contribution versus these established mechanisms remains to be quantified in human studies (51, 52).

MASLD and TI intersect at the metabolic-immune interface. Lipotoxic substances in MASLD patients induce epigenetic reprogramming in bone marrow hematopoietic progenitors via the TLR4/MyD88/NF-κB signaling axis (53, 54), leading to sustained pro-inflammatory cytokine expression in peripheral monocytes (55). Persistent LPS exposure has been documented in MASLD livers (56); this may activate macrophages through the TLR4/NF-κB pathway in a manner consistent with trained immunity, potentially driving intrahepatic inflammation and fibrosis. Animal models confirm these findings, showing that pro-inflammatory cell infiltration in the liver is associated with systemic innate immune activation (57). Whether this specifically reflects bone marrow hematopoietic reprogramming consistent with central trained immunity requires further investigation.

More importantly, TI may act as one of several “metabolic-immune bridges” that facilitate the progression from MASLD to NASH, alongside direct lipotoxic activation, ER stress, and adaptive responses (41, 58). In NASH, metabolic stress remodels macrophages toward a TI phenotype, and these “trained” cells secrete pro-fibrotic factors such as TGF-β, linking metabolic disturbances to fibrosis (59).

A critical caveat: most murine MASLD models employ short-term high-fat diets (8–16 weeks) inducing robust TI signatures, whereas human MASLD develops over decades (41). Murine and human myeloid cells differ in glycolytic reserve and epigenetic kinetics (60). Whether lipotoxic metabolites induce persistent H3K4me3 enrichment in human hematopoietic stem cells remains unproven — current evidence is indirect from peripheral monocyte assays that may reflect ongoing inflammation rather than genuine central memory (61).

### 3.3 Liver cirrhosis and TI

Liver cirrhosis is a chronic liver condition in its final stage, caused by different factors. It involves ongoing tissue damage, abnormal healing, and increasing fibrosis, which eventually causes liver failure and portal hypertension (57). Along with the usual focus on hepatocyte injury and hepatic stellate cell activation, the enduring inflammatory memory of the innate immune system, created through TI, could significantly influence the initiation and progression of cirrhosis. Clinical studies provide direct evidence for this assertion.

Clinical studies provide direct evidence for this assertion. Monocytes in the peripheral blood of cirrhotic patients secrete significantly higher levels of TNF-α, IL-6, and IL-1β upon LPS stimulation *in vitro* compared to healthy controls (62, 63). Notably, monocyte hyper-responsiveness persists for months following liver transplantation (64); although this temporal persistence is compatible with centrally encoded memory, alternative explanations (ongoing endotoxemia, immunosuppressant effects, or alloresponses) cannot be excluded (65), and persistence alone does not prove HSPC-level reprogramming (66). Its prognostic value was confirmed in a prospective cohort study, which found that baseline monocyte hyper-responsiveness was an independent predictor of the first decompensation event within one year, as well as liver transplantation or liver-related death in patients with compensated cirrhosis. This predictive value is independent of traditional liver function indicators and the severity of portal hypertension (67).

Persistent endotoxemia, resulting from intestinal bacterial translocation, is mechanistically regarded as a crucial initiating factor for the induction and maintenance of TI (47). Continuous portal LPS drives metabolic and epigenetic reprogramming in monocytes via the TLR4/MyD88/mTOR-HIF-1α axis, resulting in succinate accumulation and H3K4me3 deposition at pro-inflammatory gene promoters, as described in Section 1 (68, 69) Animal studies suggest that metabolic reprogramming may underlie persistent hepatic inflammation even after cessation of injury. In rodent fibrosis models, pharmacological inhibition of glycolysis—such as with 2-deoxyglucose—has been shown to attenuate macrophage activation and reduce collagen deposition (68), supporting the concept that a ‘metabolic memory’ in hepatic myeloid cells contributes to fibrotic progression. However, whether this phenomenon represents bona fide trained immunity at the epigenetic level remains to be formally demonstrated.

Preliminary preclinical studies indicate the potential therapeutic value of targeting critical nodes of TI. In a diet-induced NASH-related liver fibrosis mouse model, treatment with the DNA methyltransferase inhibitor 5-azacytidine or the histone methyltransferase inhibitor MI-2 effectively reversed abnormal epigenetic modifications in hepatic stellate cells and macrophages (70). While hepatic stellate cells are not innate immune cells and thus fall outside the strict definition of trained immunity, these findings illustrate that metabolic-epigenetic reprogramming logic may be shared across cell types in fibrotic liver disease. Although dedicated clinical trials in cirrhosis patients are currently lacking, indirect evidence from studies on tuberculosis vaccines, such as BCG revaccination, suggests that epigenetic reprogramming can be induced in humans for at least 3 months, thereby providing preliminary support for exploring similar intervention strategies. As discussed in Section 1.6, species-specific differences in glycolytic reserve and epigenetic kinetics (60), together with the training-tolerance spectrum (36, 71) and divergent fibrotic memory dynamics between murine models and humans (41, 72), complicate direct translation and mandate careful patient stratification.

### 3.4 Hepatocellular carcinoma and TI

HCC ranks third in cancer-related deaths worldwide, with the majority of cases, over 80%, occurring in patients with cirrhosis (73). Besides conventional risk factors like HBV/HCV infection, alcohol use, and metabolic dysfunction-associated fatty liver disease, some individuals develop HCC without going through the usual cirrhotic phase (74). This observation suggests the presence of persistent low-grade inflammation that may drive carcinogenesis. The TI hypothesis provides a novel perspective on this mechanism: when innate immune cells with long-term memory infiltrate the liver microenvironment, they may induce sustained inflammation, oxidative stress, and genomic instability, thereby facilitating the development of HCC (44, 75).

Population-based epidemiological studies offer significant insights into this association. Research involving a large group of participants showed that those with elevated baseline high-sensitivity C-reactive protein levels were at a significantly increased risk for HCC (76). Similarly, the European EPIC cohort study demonstrated that higher peripheral blood IL-6 levels were connected to an elevated risk of HCC (77). Although elevated hs-CRP and IL-6 are downstream effectors compatible with a trained immune phenotype, these acute-phase reactants lack TI specificity because they also reflect hepatocyte stress, adipose inflammation, and non-TI pathways (17, 45); notably, no study directly assessed TI-specific epigenetic marks in patient monocytes (78). Consequently, population-level data support a role for chronic inflammation in hepatocarcinogenesis but do not constitute surrogate evidence for innate immune memory.

Consistent with the systemic training described in Sections 2.3 and 4.2, low-dose gut-derived LPS trains myeloid cells that are recruited to hepatic sinusoids via the CCR2 axis, where they synergize with Kupffer cells to create a protumorigenic microenvironment (75).This microenvironment promotes HCC through multiple pathways: on one hand, sustained NF-κB signaling and NLRP3 inflammasome activation lead to oxidative DNA damage and mutation accumulation in hepatocytes, and activate hepatic stellate cells via the IL-1β/IL-17 axis, accelerating the fibrosis-cirrhosis-HCC sequence (79); on the other hand, trained Kupffer cells secrete large amounts of TGF-β1, inhibiting IFN-γ production by CD8^+^ T cells, thereby helping potentially mutated hepatocyte clones evade immune surveillance (75). Animal experiments support this: high-fat diet mice subjected to repeated low-dose LPS challenges developed sustained hepatic NLRP3 expression, lipid deposition, and pro-inflammatory monocyte infiltration (79); intervention with an IL-1 receptor antagonist significantly alleviated intrahepatic tumor progression, suggesting that blocking the excessive inflammation triggered by TI could be a novel strategy for HCC prevention (80). Caution is warranted, however, because TI is not inherently protumorigenic—BCG-induced trained immunity can enhance antitumor surveillance and reduce HCC recurrence in some models (81)—and its net effect in HCC likely depends on training dose, timing, and concurrent adaptive immunity (44).

### 3.5 Other liver diseases and TI

In addition to the major chronic liver diseases previously discussed, the impact of TI spans a wide range of other hepatic pathologies. In these contexts, TI serves as a “double-edged sword,” with its function oscillating between a primary pathogenic driver, as observed in autoimmune and alcoholic liver diseases, and a provider of crucial protective memory, as seen in specific acute viral or parasitic infections. The various functions and essential regulatory mechanisms of TI in these additional liver diseases are summarized in **Table 1**.

**Table 1:** The role and key mechanisms of TI in various liver diseases.

Disease category	Specific disease	Predominant role of TI	Key mechanisms	References
Bile Duct Diseases	Primary Biliary Cholangitis (PBC), Primary Sclerosing Cholangitis (PSC)	Pathogenic primarily	PI3K/AKT/mTOR/HIF-1α pathway, H3K4me3↑, IL-6/TNF-α↑	(3, 82);
Acute Liver Injury & Failure	Acute Liver Failure (ALF), Septic Liver Injury, Hepatic Ischemia-Reperfusion Injury (IRI)	Protective (biphasic in IRI)	Itaconate induction, MSC reprogramming, Glycolysis↑, caspase-1/11 dependent	(83–85)
Autoimmune Liver Diseases	Autoimmune Hepatitis (AIH)	Pathogenic primarily	Epigenetic reprogramming maintaining chronic inflammation	(86, 87)
Acute Viral Hepatitis	Acute HBV, Adenovirus, etc.	Protective primarily	*In utero*/early life exposure inducing non-specific antiviral memory	(88)
Alcohol-Related Liver Disease	Alcoholic Liver Disease (ALD)/Alcoholic Hepatitis	Pathogenic primarily	Kupffer cell & NKT reprogramming, TNF-α↑	(89, 90)
Drug-Induced Liver Injury	Drug-Induced Liver Injury (DILI)	Pathogenic primarily	DAMPs amplify innate immunity	(91)
Parasitic Liver Diseases	Hepatic stage malaria, Echinococcosis, etc.	Biphasic	TLR-nucleic acid pathways, CD8 help or parasite manipulation of TI	(92)

## 4 TI in periodontitis

### 4.1 Epidemiology of periodontitis and mechanisms of immune imbalance

Periodontitis is a chronic inflammatory disease caused by dental plaque bacteria, leading to the progressive destruction of tooth-supporting structures, including the gingiva, periodontal ligament, and alveolar bone, ultimately resulting in tooth mobility and loss. Globally, around 50% of adults are affected, with 10%-15% experiencing moderate to severe forms (93, 94). In addition to inadequate oral hygiene, smoking and diabetes are well-established risk factors (95). Not only is periodontitis the primary cause of adult tooth loss, but it also contributes significantly to systemic inflammation, thereby heightening the susceptibility to cardiovascular and cerebrovascular diseases and complicating diabetes management (96).

TI is increasingly recognized as one contributing mechanism to disease progression, operating alongside biofilm-induced direct tissue destruction, adaptive Th17/Treg imbalance, and genetic susceptibility factors (60). Following dysbiosis of the plaque microbiome, pathogens and their virulence factors can infiltrate the circulation through ulcerated gingival epithelium, persistently inducing epigenetic reprogramming and metabolic remodeling in myeloid cells. This process enhances the response capacity of innate immune cells over the long term (3, 97). Consequently, this mechanism is proposed to result in the overproduction of pro-inflammatory cytokines such as IL-1β, TNF-α, and IL-6 within the periodontium, which subsequently disseminate systemically. Additionally, it may prompt a lineage shift in bone marrow hematopoietic stem cells, thereby establishing a self-reinforcing “inflammatory memory” circuit (95). This feedback, if TI-driven, not only would accelerate local alveolar bone resorption but also increase systemic inflammatory load, thereby functioning as a proposed immunological node that links local periodontal pathology to systemic inflammation, potentially contributing to the disease’s chronicity and systemic impact.

### 4.2 Mechanisms of TI in periodontitis

The invasion of harmful microorganisms and the host’s immune response, notably the persistent inflammation caused by TI, contribute to the development and progression of periodontitis.By means of epigenetic reprogramming and metabolic changes, TI grants innate immune cells a durable ‘memory,’ which sustains inflammation and tissue damage locally in the periodontium and across the body.

#### 4.2.1 Local inflammation maintenance and tissue destruction

In the local periodontal environment, virulence factors like lipopolysaccharide from key pathogens such as Porphyromonas gingivalis can induce stromal cells like gingival fibroblasts to exhibit characteristic features of TI. These cells show enrichment of the activating histone mark H3K4me1 at enhancer regions of inflammatory genes at the epigenetic level, a metabolic shift favoring glycolysis, and significantly increased secretion of pro-inflammatory cytokines like IL-6 and TNF-α at the functional level (22). These changes collectively sustain the persistent inflammatory microenvironment within the periodontal pocket and promote alveolar bone resorption through mechanisms like osteoclast activation. Notably, histone methyltransferase inhibitors and PI3K pathway inhibitors have been shown experimentally to effectively reverse this TI phenotype, identifying potential therapeutic targets for clinical intervention (98).

Beyond histone modifications, periodontal pathogen-derived LPS has been shown to activate the PI3K/AKT/mTOR signaling axis in gingival fibroblasts (22); parallel mechanisms involving NLRP3-dependent innate immune reprogramming have been demonstrated in systemic models of Western diet-induced inflammation (98). Succinate accumulation has been detected in inflamed periodontal tissues (16), aligning periodontal infection with the core metabolic-epigenetic circuit characteristic of TI (detailed in Section 1.2 and 1.4). Notably, nearly all mechanistic studies on TI in periodontitis employed *in vitro* stimulation of cell lines with P. gingivalis LPS — revealing transient changes but not durable “memory” lasting weeks (22). Direct evidence for *in vivo* training of human gingival resident macrophages or hematopoietic stem cells by periodontal pathogens is lacking (99).

#### 4.2.2 Systemic inflammation and comorbidity associations

The impact of periodontitis extends far beyond the local oral cavity. By inducing durable epigenetic reprogramming in bone marrow hematopoietic stem and progenitor cells, it systemically exacerbates systemic inflammation levels, thereby closely linking to the pathogenesis of various comorbidities (9, 100). This process leads to the overproduction of functionally hyperactive myeloid cells. When these “pre-activated” cells enter the circulation, they not only exacerbate periodontal tissue destruction but also infiltrate distant organs. For example, overactivated monocytes readily differentiate into macrophages and take up lipids, a process compatible with atherogenesis. Their secreted TNF-α may also interfere with insulin signaling, worsening diabetes (101). Additionally, enzymes such as peptidylarginine deiminase from P. gingivalis citrullinate host proteins, generating autoantigens that increase rheumatoid arthritis risk (102). Therefore, by systemically altering the host immune state through TI mechanisms, periodontitis plays a key driving role in the pathological link to comorbidities such as cardiovascular disease, diabetes, and rheumatoid arthritis. Multiple lines of evidence support the systemic training role of periodontitis. In a landmark study, Li et al. demonstrated that periodontitis in mice induces persistent epigenetic reprogramming of hematopoietic stem and progenitor cells (HSPCs) in the bone marrow, characterized by H3K4me3 enrichment at promoters of pro-inflammatory genes, and adoptive transfer of these trained HSPCs into naïve recipients recapitulated systemic inflammation (103). In humans, circulating monocytes from periodontitis patients exhibit sustained hyper-responsiveness to TLR2 and TLR4 ligands following successful local therapy, suggesting that persistent innate immune remodeling may have been established (9). Moreover, eradication of P. gingivalis by targeted antibiotic therapy partially reverses the trained phenotype in animal models, indicating that TI is at least modifiable (103).

#### 4.2.3 Persistently enhanced neutrophil function

Neutrophils, as the initial defense in the periodontium, show notable functional changes during TI. Research indicates that neutrophils in the peripheral blood of periodontitis patients have a notably increased ability to form neutrophil extracellular traps and produce reactive oxygen species (104). This hyperfunctional state is closely associated with the accumulation of TI-related epigenetic modifications, placing the cells in a “primed” state. Although this may enhance pathogen clearance, overactivated neutrophils inevitably exacerbate immune-mediated damage to periodontal connective tissue and bone by releasing large amounts of proteolytic enzymes and ROS, thus creating a positive feedback loop of progressively worsening tissue destruction. It should be noted, however, that the concept of trained immunity in short-lived neutrophils is still evolving, and the contribution of epigenetic memory versus continuous bone marrow education remains to be clarified.

#### 4.2.4 Core molecular evidence for TI in periodontitis

In summary, converging lines of evidence support TI as a plausible mechanism in periodontitis: (i) P. gingivalis LPS induces durable H3K4me3 and H3K27ac enrichment at enhancer regions of IL-6 and TNF-α in gingival fibroblasts and monocytes (105); (ii) this epigenetic remodeling is mediated through the PI3K/AKT/mTOR/HIF-1α metabolic axis, with increased glycolysis and succinate accumulation as functional hallmarks (18, 102); (iii) trained cells from periodontitis patients or animal models show enhanced secondary responses to unrelated stimuli (e.g., low-dose LPS or F. nucleatum), consistent with the operational definition of TI (100); and (iv) adoptive transfer of trained HSPCs from periodontitis-bearing mice recapitulates systemic hyper-inflammation in naïve recipients, supporting a causal interpretation (103). Collectively, these data suggest that periodontitis operates as not merely a local destructive disease but potentially a systemic immune training stimulus.

### 4.3 Therapeutic prospects and translational significance

The introduction of the TI concept offers a new paradigm for periodontitis prevention and treatment, moving beyond the traditional focus on “plaque control.” Current intervention strategies targeting TI primarily revolve around reversing the core epigenetic and metabolic reprogramming. For example, histone methyltransferase inhibitors (e.g., targeting MLL1) and PI3K/Akt/mTOR pathway inhibitors have been shown in *in vitro* models to effectively dismantle the TI phenotype in periodontal-derived cells (e.g., fibroblasts) and reduce their pro-inflammatory cytokine secretion (98). At the metabolic level, using glycolysis inhibitors (e.g., 2-deoxyglucose) or mitochondrial function modulators holds promise for reversing the Warburg effect implicated in TI maintenance, thereby potentially attenuating sustained inflammatory output.

A strategy with greater translational potential lies in the re-evaluation and repurposing of existing therapies. Basic research and clinical observations suggest that effective non-surgical periodontal treatment not only removes the local plaque biofilm, eliminating the initial trigger for TI, but may also indirectly promote the “resetting” of the epigenetic state of immune cells by lowering systemic inflammation levels. Furthermore, some drugs used for systemic diseases, such as metformin and statins, are hypothesized to potentially alleviate the TI state due to their combined metabolic-regulating and mild anti-inflammatory properties (101), although their direct capacity to reverse trained immunity in humans remains to be validated.

However, translating the theory of TI into clinical practice still faces challenges. The primary issue is the lack of specific biomarkers to assess the “trained” state of periodontal tissues or circulating immune cells *in vivo*. Future research should focus on discovering and validating markers detectable in clinical settings, such as specific histone modification profiles, metabolite profiles, or functional immune indices. Simultaneously, precise interventions targeting TI must carefully balance the potential risks of systemic immunomodulation. How to attenuate harmful inflammatory memory while preserving the body’s normal anti-infective defense capacity is a critical balance that future drug development must prudently consider.

Furthermore, clinical data on periodontal treatment reversing TI are conflicting (106). Some studies show scaling and root planing improves liver enzymes in MASLD; others report no reduction in circulating IL-6 or monocyte responsiveness (107, 108). This suggests established TI may be resistant to local intervention alone, and patient stratification by baseline TI status (**Table 2b**) may be necessary for future trials (110). For example, patients with high-reversibility metabolic markers (elevated succinate, early-stage MASLD) may respond to periodontal therapy alone, whereas those with low-reversibility epigenetic marks (persistent H3K4me3, advanced fibrosis) may require combined epigenetic modulator and periodontal intervention (41).

## 5 The liver-gut-immune-oral axis: TI-mediated comorbidity mechanisms

### 5.1 Conceptual framework

The “Liver-Gut-Immune-Oral Axis” is proposed here as a hypothetical pathophysiological network involving the liver, gut, immune system, and oral cavity, interconnected through systemic inflammation and immune memory. TI is hypothesized as the core mechanism enabling the systemic propagation and maintenance of “inflammatory memory” within this axis. To date, this model is constructed by integrating findings from separate experimental systems, genetic models, and associative clinical observations; no single study has simultaneously captured all four nodes of the axis within one cohort or experimental design.

### 5.2 Forward pathway: liver disease → systemic TI → periodontitis

**Figure 1:**
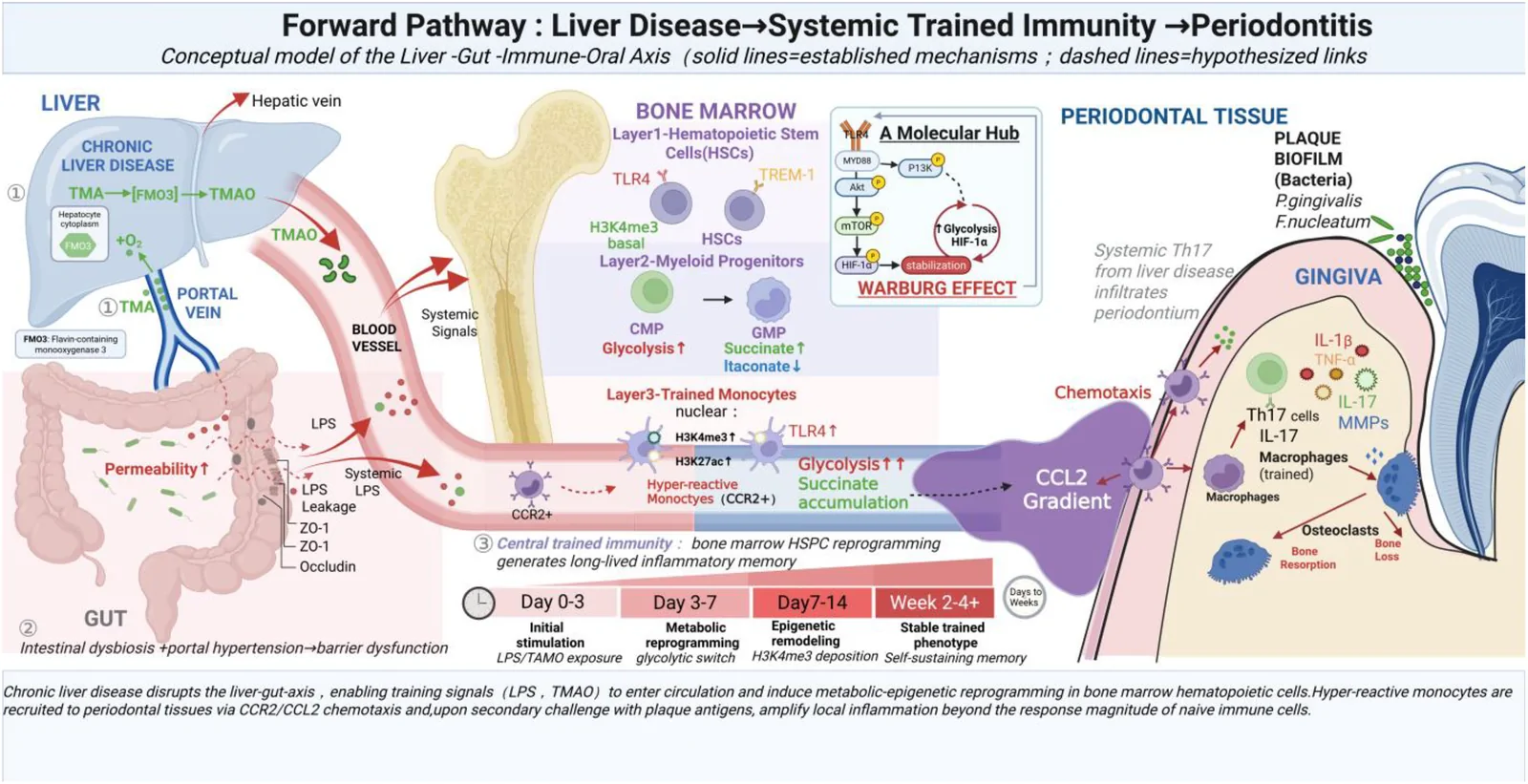
Conceptual model of the forward pathway in the liver-gut-immune-oral axis. Created in BioRender. Li, X. (2026) https://BioRender.com/acv33ws.

A proposed primary mechanism through which chronic liver disease may contribute to periodontitis is its potential to induce a systemic inflammatory state, possibly mediated by TI. This process initiates with the dysfunction of the liver-gut axis (**Figure 1**). Hepatic insufficiency and portal hypertension work synergistically to compromise the intestinal barrier, resulting in bacterial translocation and persistent low-grade endotoxemia. This condition permits PAMPs, such as lipopolysaccharide, to continuously enter the bloodstream (47). Simultaneously, circulating metabolites such as trimethylamine N-oxide (TMAO), which can be generated through hepatic metabolism and have been shown to enhance trained immunity in experimental models (39), may contribute to a systemic pool of training signals together with gut-derived PAMPs; however, the specific contribution of hepatocyte-derived TMAO to bone marrow reprogramming in chronic liver disease remains to be established.

A principal inferred target of these systemic signals is the bone marrow hematopoietic system (**Figure 1**). Persistent stimulation via TLR4/MyD88 and related pathways has been shown to induce metabolic-epigenetic reprogramming of HSPCs and myeloid progenitors in experimental systems, consistent with central TI as detailed in Sections 1 and 2 (99, 111).This “central training” results in sustained production of hyper-reactive monocytes, whose enhanced responsiveness can persist even after liver transplantation (47, 63).

Following systemic training, immune cells are specifically recruited to periodontal tissues. They migrate to the gingiva through high expression of the chemokine receptor CCR2, which responds to chemokines such as CCL2 released at periodontal lesion sites (28). Once in the gingiva, dental plaque microbial antigens such as LPS would serve as a secondary stimulus, activating the pre-established epigenetic program in trained macrophages. This activation is expected to amplify secretion of IL-1β, TNF-α, IL-6, and matrix metalloproteinases. Concurrently, systemic Th17 cells associated with liver disease infiltrate the periodontium, with their secreted IL-17 working synergistically with the aforementioned factors to strongly activate osteoclasts and worsen connective tissue destruction, ultimately leading to the onset or exacerbation of periodontitis (95).

Several caveats apply. First, this forward pathway is synthesized from independent studies; although individual segments are supported—e.g., endotoxemia in cirrhosis (47), monocyte hyper-responsiveness ex vivo (62, 67), and CCR2-dependent recruitment in periodontitis models (27)—no single investigation has simultaneously tracked liver-gut barrier disruption, bone marrow HSPC reprogramming with TI-consistent epigenetic signatures, and trained monocyte amplification in periodontal tissues (99, 112, 113). Second, human evidence for central HSPC training remains indirect (114, 115). Third, persistent endotoxemia may prime circulating monocytes without bone marrow reprogramming—an alternative mechanism with distinct therapeutic implications (barrier restoration versus epigenetic modulation) (112, 116, 117).

Chronic liver disease may predispose to periodontitis via systemic trained immunity (TI). Liver-gut axis disruption enables training signals (LPS, TMAO) to enter circulation, potentially inducing metabolic-epigenetic reprogramming in bone marrow hematopoietic cells (enhanced glycolysis, H3K4me3 deposition). Hyper-reactive monocytes may be recruited to periodontal tissues via the CCR2/CCL2 axis and, upon secondary challenge with plaque antigens, amplify local inflammation. Solid lines denote mechanisms supported by independent studies; dashed lines denote hypothetical connections requiring integrative validation.

### 5.3 Reverse pathway: periodontitis → systemic TI → liver disease

Periodontitis, characterized as a chronic infectious condition, facilitates the entry of key pathogens, such as Porphyromonas gingivalis and Fusobacterium nucleatum, along with their virulence factors, including LPS, gingipains, and peptidylarginine deiminase. Additionally, locally overproduced inflammatory cytokines, such as IL-1β, TNF-α, IL-6, and IL-17, can penetrate the circulation through ulcerated gingival epithelium. Once in the bloodstream, these elements disseminate systemically and exert significant effects on distant organs, particularly the liver (105). This reverse pathway is hypothesized to employ TI as a fundamental mechanism, transforming localized oral chronic inflammation into persistent systemic inflammatory memory, which ultimately exacerbates liver pathology.

Periodontal-derived PAMPs and DAMPs have been shown to exert systemic effects on bone marrow HSPCs in murine models, where they induce epigenetic and metabolic reprogramming consistent with central TI (see Sections 1 and 3 for detailed mechanisms). Consequently, the bone marrow may become biased towards the generation of pro-inflammatory monocytes and neutrophils; these “trained” myeloid cells are continuously released into the peripheral blood, establishing a long-term hyper-responsive state. Even when local infections are controlled, this immune memory at the bone marrow level has been reported to persist for months, potentially acting as a remote contributor to systemic inflammation (112).

Circulating trained monocytes are recruited to the liver sinusoids via the CCR2/CCL2 axis, where they rapidly differentiate into pro-inflammatory macrophages. These macrophages synergize with resident Kupffer cells, demonstrating enhanced NLRP3 inflammasome activity, increased reactive oxygen species (ROS) production, and elevated pro-inflammatory cytokine secretion, which directly exacerbates hepatocyte lipotoxic injury, oxidative stress, and the remodeling of the inflammatory microenvironment. In addition to trained immunity-mediated inflammation, periodontal pathogens may exacerbate liver disease through distinct mechanisms; for instance, P. gingivalis has been shown to promote MASLD progression via ferroptosis (99). Trained myeloid cells may act synergistically with such pathways by sustaining intrahepatic inflammatory signaling, though their relative contribution remains to be quantified.

**Figure 2:**
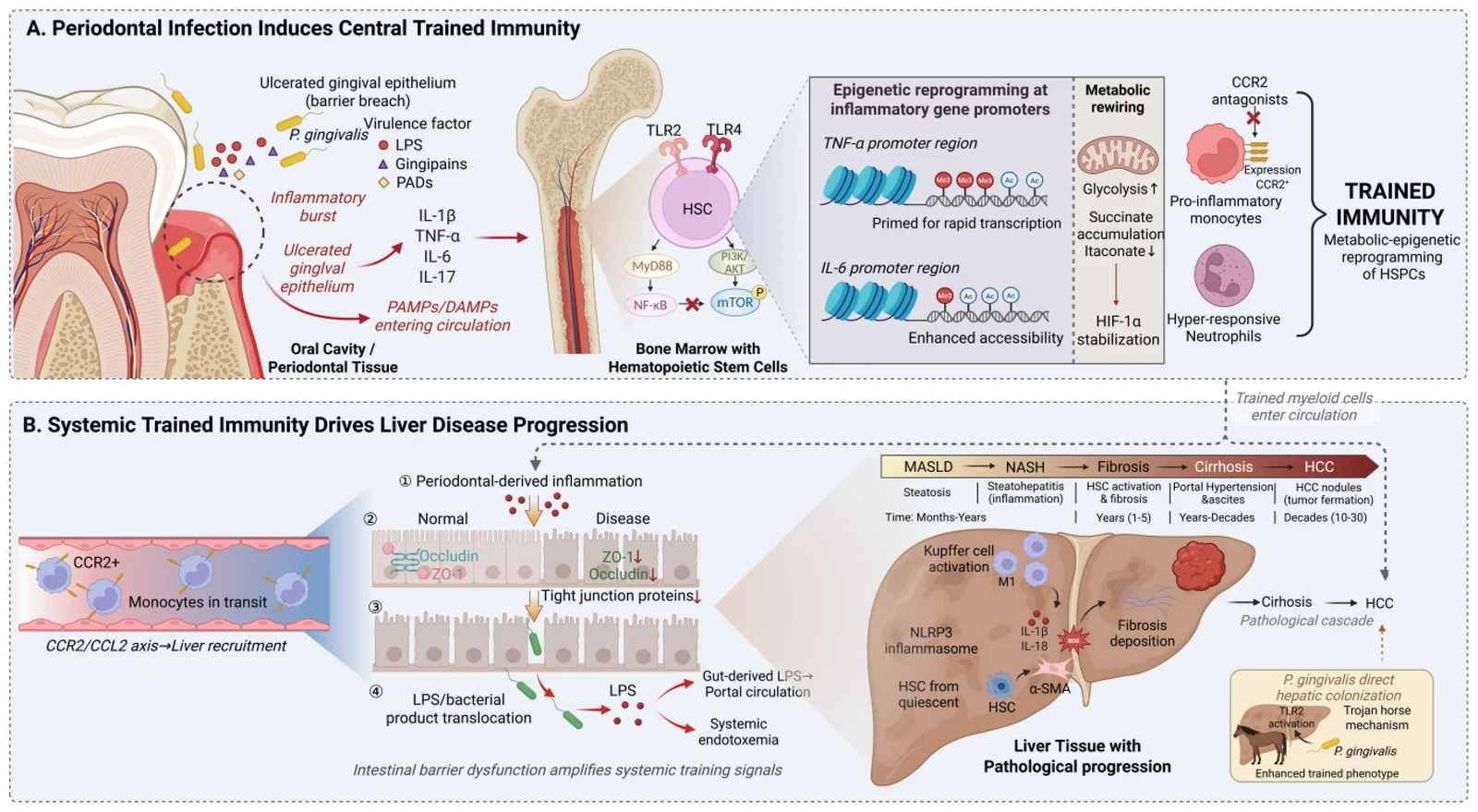
Conceptual model of the reverse pathway in the liver-gut-immune-oral axis. Created in BioRender. Li, X. (2026) https://BioRender.com/gz9290p.

Simultaneously, periodontitis-induced persistent systemic inflammation has been associated with disruption of intestinal epithelial tight junctions (decreased ZO-1, occludin expression) and increased intestinal permeability. This permeability increase may enable more gut-derived LPS and bacterial products to enter the blood and liver, adding to periodontal-derived stimuli and potentially establishing a positive feedback loop that could reinforce the TI state in bone marrow and hepatic myeloid cells, with potential consequences for liver fibrosis, cirrhosis, and HCC progression (**Figure 2**) (99). Epidemiological evidence shows that patients with severe periodontitis have a significantly higher risk of MASLD/NASH, and periodontal treatment can partially improve liver enzyme levels and steatosis severity, suggesting a clear causal association (7). Furthermore, specific pathogens like P. gingivalis can directly colonize the liver via the bloodstream or a “Trojan horse” mechanism; its gingipains activate TLR2 signaling, promoting Kupffer cell M1 polarization and reinforcing the TI phenotype. Periodontal-derived citrullinated proteins can also act as autoantigens, triggering aberrant adaptive immune responses within the liver, further amplifying injury (103). It is important to note that not all liver-periodontitis associations require TI as an explanatory mechanism (118, 119). Direct effects—including transient bacteremia during chewing or toothbrushing, periodontal pathogen translocation to the liver via the portal or systemic circulation, and shared risk factors such as smoking, diabetes, and diet—may account for part of the observed comorbidity independent of long-term innate immune memory (98, 120). The relative contribution of TI versus these acute/transient effects remains a critical open question for future research (121).

As with the forward pathway, the reverse pathway must be recognized as a conceptual construct derived from separate lines of investigation. Current evidence demonstrates that (i) periodontitis induces epigenetic changes in murine HSPCs (103), (ii) periodontal treatment modestly improves liver enzymes in some but not all clinical studies (7), and (iii) P. gingivalis can be detected in human liver tissue (99). However, no single study has yet linked periodontitis-induced HSPC reprogramming directly to accelerated liver disease progression in the same animal or human cohort by prospectively tracking trained cell migration and hepatic outcomes (119). Integrative studies combining periodontal intervention, bone marrow assessment, and liver histology or imaging are urgently needed (120, 121).

The reverse pathway concept thus highlights periodontitis’s potential role as an initiator of systemic inflammation, proposing that, through the durable mechanism of TI, chronic oral infection could remotely drive liver disease progression (122, 123). While a solid mechanistic basis exists for each segment of this route, the integrated causal pathway awaits formal demonstration (121). This provides a compelling rationale for clinical strategies like “protecting the liver by managing the mouth” in multi-disease co-management. Future targeting of TI (**Table 2a**) or implementing standard periodontal interventions (**Table 2b**) holds promise for effectively breaking this vicious cycle and improving liver disease prognosis, contingent upon validation of the proposed axis in prospective human studies (17). Patient stratification by biomarker-defined reversibility status (**Table 2b**) will be critical for trial design (17).

**Table 2A:** Clinical and translational targets in liver-periodontitis comorbidity.

Dimension	Target class	Intervention	Evidence source	Key uncertainty
Initiating signals	Gut barrier restoration	Probiotics/prebiotics	(39, 47)	Lack of standardized strains and dosing regimens
Initiating signals	Periodontal pathogen clearance	Non-surgical periodontal therapy	RCT (7)	Degree of systemic TI memory reversal not quantified
Central immune memory	mTOR inhibition	Rapamycin	*In vitro*/animal (25, 124)	Infection risk in cirrhotic patients; contraindicated in decompensated disease
Central immune memory	Glycolysis inhibition	2-deoxyglucose	Animal models (68)	Systemic metabolic side effects; poor selectivity
Central immune memory	Histone methyltransferase inhibition	MI-2, MLL1 inhibitors	Animal (70)	Off-target chromatin alterations; risk of irreversible epigenetic changes
Effector phase	CCR2/CCL2 blockade	CCR2 antagonists	Animal (28, 79)	Impaired normal monocyte recruitment and host defense
Effector phase	IL-1R blockade	Anakinra	Animal (80)	Infection risk in cirrhotic patients; efficacy untested in liver-periodontitis comorbidity
Effector phase	MMP inhibition	MMP inhibitors	Preclinical	Lack of tissue specificity; may impair physiological remodeling

**Table 2B:** Biomarkers and intervention windows for trained immunity in liver-periodontitis comorbidity.

Biomarker class	Specific indicator	Disease stage applicability	Reversibility	Representative references
Metabolic markers	Serum succinate, lactate	Early-stage (compensated cirrhosis, MASLD)	High	(19, 67)
Metabolic markers	TMAO	MASLD/NASH, advanced fibrosis	Moderate	(39, 109)
Epigenetic markers	Circulating monocyte H3K4me3 enrichment	All stages	Moderate (persists post-transplant)	(21, 69)
Functional markers	Ex vivo LPS-stimulated TNF-α/IL-6 production	All stages	Low (advanced fibrosis/HCC)	(62, 67)
Local periodontal markers	Gingival crevicular fluid VAP-1, TSP-1	Active periodontitis	High (improves after periodontal therapy)	(109)
Systemic inflammatory markers	Serum IL-6, hs-CRP	All stages	Low (nonspecific; reflects multiple pathways)	(17, 76)

Nevertheless, contradictory findings exist. Randomized controlled trials of periodontal therapy show only modest and inconsistent improvements in liver enzymes, suggesting TI induced by periodontitis may be partially irreversible or that gut microbiome/genetics dominate liver disease progression (123, 125). Most animal models inject P. gingivalis intravenously or via gavage at high doses, which does not mimic chronic human periodontitis. Whether long-standing periodontitis truly reprograms human bone marrow progenitors or merely induces transient activation remains unresolved (17, 120).

Periodontitis may exacerbate liver disease through systemic trained immunity (TI): periodontal pathogens and their virulence factors enter the circulation and may induce epigenetic reprogramming and metabolic rewiring of hematopoietic stem cells in the bone marrow. This process could generate hyper-inflammatory myeloid cells that migrate to the liver, where they may synergize with gut-derived endotoxemia to accelerate the pathological transition from MASLD to hepatocellular carcinoma. Solid lines denote independently validated mechanisms; dashed lines denote hypothetical links awaiting prospective confirmation.

To systematically summarize the complex mechanisms by which trained immunity mediates liver-periodontitis comorbidity, we present three comprehensive tables. **Table 3** delineates the core mechanistic dimensions of trained immunity-–encompassing metabolic reprogramming, epigenetic remodeling, central signaling hubs, cellular players, and key soluble mediators-–and illustrates their specific manifestations in both liver disease and periodontitis. **Table 2a** translates these mechanistic insights into clinical therapeutic targets across the Liver-Gut-Immune-Oral Axis, from initiating signals through central immune memory to effector phases, with explicit evidence grading and safety considerations. **Table 2b** maps candidate biomarkers for trained immunity status by disease stage and reversibility potential, providing a framework for patient stratification and intervention timing.

**Table 3:** Core mechanisms of trained immunity in liver disease and periodontitis.

Mechanistic dimension	Core driver	Liver disease established/emerging/hypothesized	Liver: evidence grade & source	Periodontitis established/emerging/hypothesized	Perio: evidence grade & source	Cross-disease conservation	Representative references
Metabolic Reprogramming	Aerobic glycolysis (Warburg effect); succinate accumulation	Established: Shift from OXPHOS to aerobic glycolysis documented in chronic liver disease; elevated lactate production in hepatic models; glycolysis & succinate stabilize HIF-1α promoting IL-1β & TNF-α.	★★★ [Mouse][*In vitro*][Clinical cohort] (16, 17)	Established:P. gingivalis-LPS drives mTOR/HIF-1α shifting OXPHOS→glycolysis in periodontal tissues; confirmed in multiple models.	★★★ [Human][Mouse][*In vitro*] (13, 15)	✓ Validated	(16, 18–20)
		Emerging: Succinate accumulation specifically stabilizing HIF-1α in hepatic trained immunity context; single-center observations.	★★☆ [Mouse][*In vitro*] (13)	Emerging: Gingival fibroblasts exhibit ↑glycolysis & lactate production uponP. gingivalischallenge; preliminary findings.	★★☆ [Human][*In vitro*] (15)	~ Partial	
		Hypothesized: Warburg effect as adirectdriver of hepatic trained immunity program (beyond association); lacks causal validation.	☆☆☆ [*In vitro*] (15)	Hypothesized: Periodontal Warburg effect directly links to systemic trained immunity propagation; no integrative study confirms.	☆☆☆ [Mouse] (17)	? Unproven	
Epigenetic Remodeling	H3K4me3, H3K27ac enrichment at promoters of inflammatory genes	Established: In chronic hepatitis/cirrhosis, persistent endotoxemia induces H3K4me3 enrichment at TNF-α/IL-6 promoters in monocytes/macrophages; this markpersists after liver transplantation.	★★★ [Human][Clinical cohort][Ex vivo] (18, 47)	Established: Periodontal pathogens induce H3K4me3 at enhancers of inflammatory genes in gingival fibroblasts/myeloid cells; replicated across studies.	★★★ [Human][*In vitro*] (19)	✓ Validated	(21, 22, 69, 126)
		Emerging:H3K27ac enrichment patterns in liver-trained monocytes; HDAC-mediated chromatin accessibility changes in hepatic context.	★★☆ [Mouse][*In vitro*] (84)	Emerging: HDAC7-mediated changes enhance gene accessibility in periodontal myeloid cells; novel mechanistic insight.	★★☆ [*In vitro*] (84)	~ Partial	
		Hypothesized: Epigenetic marks (H3K4me3/H3K27ac) as predictive biomarkers for post-transplant immune status; not prospectively validated.	☆☆☆ [Human][Clinical cohort] (18)	Hypothesized: Periodontal pathogen-induced epigenetic reprogramming in gingiva directly modifies immune gene accessibility in distal organs (liver).	☆☆☆ [Mouse] (47)	? Unproven	
Central Hub Signaling	mTOR–HIF-1α axis integrates metabolic state and inflammatory signals	Established: Portal LPS/P. gingivalis-LPS activates mTOR via TLR4/MyD88 or PI3K/AKT, stabilizing HIF-1α; this hub integrates metabolic state & drives inflammatory gene expression and NLRP3 activation.	★★★ [Mouse][Human][*In vitro*] (21, 85)	Established:P. gingivalisactivates PI3K/AKT/mTOR pathway in periodontitis; confirmed in multiple independent studies.	★★★ [Human][*In vitro*][Mouse] (23, 85)	✓ Validated	(25, 27, 124)
		Emerging:P. gingivalis-LPS specifically activates mTOR via PI3K/AKT in liver macrophages; limited to single-center studies.	★★☆ [Mouse][*In vitro*] (23)	Emerging:mTOR inhibition (e.g., rapamycin) blocks trained immunity-like responses in periodontitis models.	★★☆ [Mouse][*In vitro*] (23)	~ Partial	
		Hypothesized: NLRP3 inflammasome activation isentirelydependent on mTOR/HIF-1α axis in hepatic trained immunity; redundancy with other pathways not excluded.	☆☆☆ [*In vitro*] (21)	Hypothesized:mTOR inhibitors as routine adjunctive therapy for periodontitis to interrupt trained immunity; no clinical trial data in this context.	☆☆☆ [Mouse] (85)	? Unproven	
Cellular Players	Monocytes/macrophages (peripheral); HSPCs (central)	Established: Intrahepatic macrophages (Kupffer cells) & recruited monocytes show sustained pro-inflammatory phenotype; confirmed in human cirrhosis cohorts.	★★★ [Human][Mouse][Clinical cohort] (24, 78)	Established: Circulating monocytes hyper-responsive to secondary challenge in periodontitis patients; replicated ex vivo.	★★★ [Human][Ex vivo][Clinical cohort] (24, 78)	✓ Validated	(28, 75, 104)
		Emerging: Liver-derived signals reprogram bone marrow HSPCs to generate hyper-responsive myeloid cells; adoptive transfer studies in mice.	★★☆ [Mouse] (52)	Emerging: Gingival macrophages/neutrophils exhibit primed phenotype: ↑NETs/NETosis & ROS; preliminary human data.	★★☆ [Human][*In vitro*] (52)	~ Partial	
		Hypothesized: Liver-derived signalsdirectlyreprogram HSPCs to worsen periodontitis —not been empirically demonstrated in a single integrative study.	☆☆☆ [Mouse] (24, 52)	Hypothesized: Trained immune cells from gingiva directly migrate to liver sinusoids and drive hepatic inflammation; lacks direct tracking evidence.	☆☆☆ [Mouse] (78)	? Unproven	
Key Soluble Mediators	IL-17, TGF-β; IL-1β, TNF-α, IL-6	Established: Trained macrophages secrete ↑IL-1β/TNF-α, activating hepatic stellate cells; sustained TGF-β promotes fibrotic progression (NASH→cirrhosis).	★★★ [Human][Mouse][Clinical cohort] (40, 70)	Established: Local overproduction of IL-1β, TNF-α, IL-6 drives osteoclast activation & connective tissue breakdown in periodontitis.	★★★ [Human][Clinical cohort][*In vitro*] (55, 70)	✓ Validated	(59, 79, 95)
		Emerging: IL-17 axis contributes to hepatic fibrosis via IL-1β/IL-17 crosstalk; early-stage mechanistic studies.	★★☆ [Mouse][*In vitro*] (55)	Emerging: Th17-derived IL-17 synergizes with macrophage cytokines to amplify bone resorption; emerging mechanistic link.	★★☆ [Mouse][Human] (55)	~ Partial	
		Hypothesized: TGF-β functions as adirecteffector molecule of trained immunity program rather than a secondary fibrogenic mediator.	☆☆☆ [*In vitro*] (40)	Hypothesized: Periodontal-derived cytokines (IL-1β, TNF-α) directly train bone marrow HSPCs via circulation; no direct evidence of HSPC exposure.	☆☆☆ [Mouse] (40, 70)	? Unproven	

## 6 Conclusion and prospects

TI serves as a critical systemic bridge connecting chronic liver disease and periodontitis through the “Liver-Gut-Immune-Oral Axis.” In this review, we propose the Liver-Gut-Immune-Oral Axis as a synthetic, testable framework to conceptualize the comorbidity of chronic liver disease and periodontitis. We do not claim to have established a unified causal mechanism; rather, we integrate mechanistic and epidemiological evidence to generate hypotheses for future investigation. The central premise—that innate immune memory propagates inflammation across anatomically distant sites via bone marrow reprogramming—derives primarily from independent experimental systems and requires validation through longitudinal cohort studies incorporating simultaneous multi-omics profiling of liver, gut, oral, and hematopoietic compartments.

Our principal contributions are threefold. First, we advance a multi-organ conceptual scaffold that moves beyond single-disease silos, while explicitly delineating where evidence ends and extrapolation begins. Second, we identify the mTOR--HIF-1α--glycolysis axis and histone-modifying enzymes as candidate druggable nodes (**Table 2a**) that, if the proposed axis is validated, could disrupt inter-organ inflammatory reinforcement. Third, we highlight critical evidence gaps-–particularly the lack of integrated studies tracking trained cells from origin to distal effector sites-–and propose biomarker-stratified approaches (**Table 2b**) to guide future experimental and clinical trial design.

Nevertheless, substantial challenges impede clinical translation. Most critically, the Liver-Gut-Immune-Oral Axis remains a conceptual framework that lacks direct integrative validation: no single prospective cohort study has yet simultaneously characterized all four nodes of this axis with multi-omics resolution. The causal evidence chain-–from organ-specific insult through bone marrow training to remote organ exacerbation-–requires validation through large-scale prospective cohorts incorporating longitudinal multi-omics profiling, ideally with simultaneous sampling of liver, gut, oral, and blood compartments. Mechanistic heterogeneity across etiologies-–viral, metabolic, and alcoholic-–demands single-cell resolution to determine whether tissue-specific or disease-specific epigenetic signatures exist. Critical unknowns include the reversibility of inflammatory memory across disease stages and the identification of optimal intervention windows, while standardized non-invasive biomarkers for assessing individual TI status are undeveloped, hindering patient stratification and therapeutic monitoring.

A paramount priority for future research is to design prospective, multi-cohort, multi-omics studies that simultaneously sample the oral cavity, gut, blood, and liver compartments to empirically validate — or falsify — the proposed causal chain of the Liver-Gut-Immune-Oral Axis. Currently, no single study has directly tracked trained cells from bone marrow to distant effector sites across this axis in humans or animals.

To close the causal chain of the proposed axis, we specifically advocate for integrative study designs: (i) parabiosis or photoconversion lineage-tracing to track trained cells from bone marrow to periodontal tissue or liver; (ii) human cohorts with simultaneous oral microbiome profiling, bone marrow aspirate epigenomic analysis, and liver imaging; and (iii) interventional trials stratified by baseline TI biomarker status to distinguish peripheral priming from central training.

Beyond these mechanistic validations, future research priorities should focus on four directions. First, dynamic multi-omics characterization of liver disease-periodontitis comorbidity cohorts to map the evolutionary landscape of this interaction axis. Second, precision intervention strategies targeting metabolic-epigenetic nodes, with careful evaluation of impacts on systemic immune homeostasis. Third, drug repurposing investigations assessing metformin, statins, and epigenetic modulators for their capacity to reset trained states, with rigorous phase I/II biomarker endpoints (e.g., H3K4me3 enrichment, ex vivo monocyte responsiveness) rather than solely clinical outcomes, to establish proof-of-mechanism before large-scale efficacy trials. Fourth, rigorous clinical validation of periodontal treatment as a non-pharmacological approach to modulate systemic inflammatory memory and improve hepatic outcomes. Ultimately, targeting TI offers a paradigm shift capable of overcoming organ-specific therapeutic barriers and achieving integrated prevention for chronic inflammatory diseases.

Several limitations warrant explicit acknowledgment. Chronic liver diseases are etiologically heterogeneous — viral, metabolic, and alcohol-related — each may engage distinct training pathways and reversibility, yet cross-etiology comparisons are lacking. Inter-individual variability in TI responses (age, genetics, microbiome) remains uncharacterized. Longitudinal human studies tracking TI markers over disease progression or treatment are virtually absent. Alternative explanations (e.g., peripheral monocyte priming without bone marrow reprogramming) have not been rigorously excluded. Addressing these gaps requires multi-cohort, longitudinal, multi-omics investigations.

## Glossary

AIH: Autoimmune Hepatitis
ALD: Alcoholic Liver Disease/Alcoholic Hepatitis
CHB: Chronic Hepatitis B
CHC: Chronic Hepatitis C
CCR2/CCL2: Chemokine (C-C motif) receptor 2/C-C motif chemokine ligand 2
CRP: C-reactive protein
DAMPs: Damage-associated molecular patterns
DILI: Drug-Induced Liver Injury
F. nucleatum: Fusobacterium nucleatum
HCC: Hepatocellular Carcinoma
HDAC7: Histone deacetylase 7
HIF-1α: Hypoxia-inducible factor 1-alpha
HSPCs: Hematopoietic stem and progenitor cells
H3K4me3/H3K27ac: Histone H3 lysine 4 trimethylation/Histone H3 lysine 27 acetylation
IL-1β/IL-6/IL-17: Interleukin-1β/Interleukin-6/Interleukin-17
IRI: Ischemia-Reperfusion Injury
LPS: Lipopolysaccharide
MLL1: Mixed-lineage leukemia 1 (histone methyltransferase)
MMPs: Matrix metalloproteinases
MSC: Mesenchymal stem cell
MyD88: Myeloid differentiation primary response 88
mTOR: Mechanistic target of rapamycin
MASLD: Metabolic dysfunction-associated steatotic liver disease (formerly NAFLD)
NASH: Non-alcoholic steatohepatitis
NF-κB: Nuclear factor kappa-light-chain-enhancer of activated B cells
NLRP3: NOD-like receptor family pyrin domain containing 3
NETs: Neutrophil extracellular traps
PBC: Primary biliary cholangitis
PAMPs: Pathogen-associated molecular patterns
P. gingivalis: Porphyromonas gingivalis
PPAD: Peptidylarginine deiminase
PI3K/AKT: Phosphoinositide 3-kinase/Protein kinase B
PSC: Primary sclerosing cholangitis
ROS: Reactive oxygen species
TGF-β: Transforming growth factor-beta
Th17: T helper 17 cell
TI: Trained immunity
TLR2/TLR4: Toll-like receptor 2/Toll-like receptor 4
TMAO: Trimethylamine N-oxide
TNF-α: Tumor necrosis factor-alpha
TREM-1: Triggering receptor expressed on myeloid cells 1
TSP-1: Thrombospondin-1
VAP-1: Vascular adhesion protein-1
ZO-1: Zonula occludens-1
2-DG: 2-deoxyglucose
5-Aza: 5-azacytidine.
