# Correction: Trained immunity as a systemic bridge: the liver-gut-immuneoral axis in the comorbidity of chronic liver disease and periodontitis

**DOI:** 10.3389/fimmu.2026.1937952

**Published:** 2026-08-06

**Authors:** Xiang Li, Guodong Lv, Daolang Yuan, Jun Hu, Wenrong Lou, Yulin Tu, Jing Zhou, Rong Qin

**Affiliations:** 1 Kunming Medical University, Kunming, Yunnan, China; 2 Calmette Hospital Affiliated to Kunming Medical University, Kunming, Yunnan, China; 3 Department of Otorhinolaryngology, Yan’an Hospital Affiliated to Kunming Medical University, Kunming, Yunnan, China; 4 Key Laboratory of Engineering and Translational Application of Cell Derivatives of Yunnan Province, Kunming, Yunnan, China; 5 Department of Stomatology, Yan’an Hospital Affiliated to Kunming Medical University, Kunming, Yunnan, China; 6 Department of Gastroenterology, Yan’an Hospital Affiliated to Kunming Medical University, Kunming, Yunnan, China

**Keywords:** Chronic liver disease, comorbidity, liver-gut-immune-oral axis, periodontitis, trained immunity

There was a mistake in **Figure 1**, and **Figure 2** as published. After the article was published, our internal team review revealed that, during the final manuscript preparation, we mistakenly uploaded files from an early AI-generated layout draft folder to the submission system, instead of the final high-resolution vector graphics that our team had created on the BioRender platform. The AI draft was used solely for internal preliminary brainstorming of the pathway framework and was never intended for formal publication. This error was purely a file-version submission mistake, and there was no intention to conceal the use of AI.

**Figure 1:**
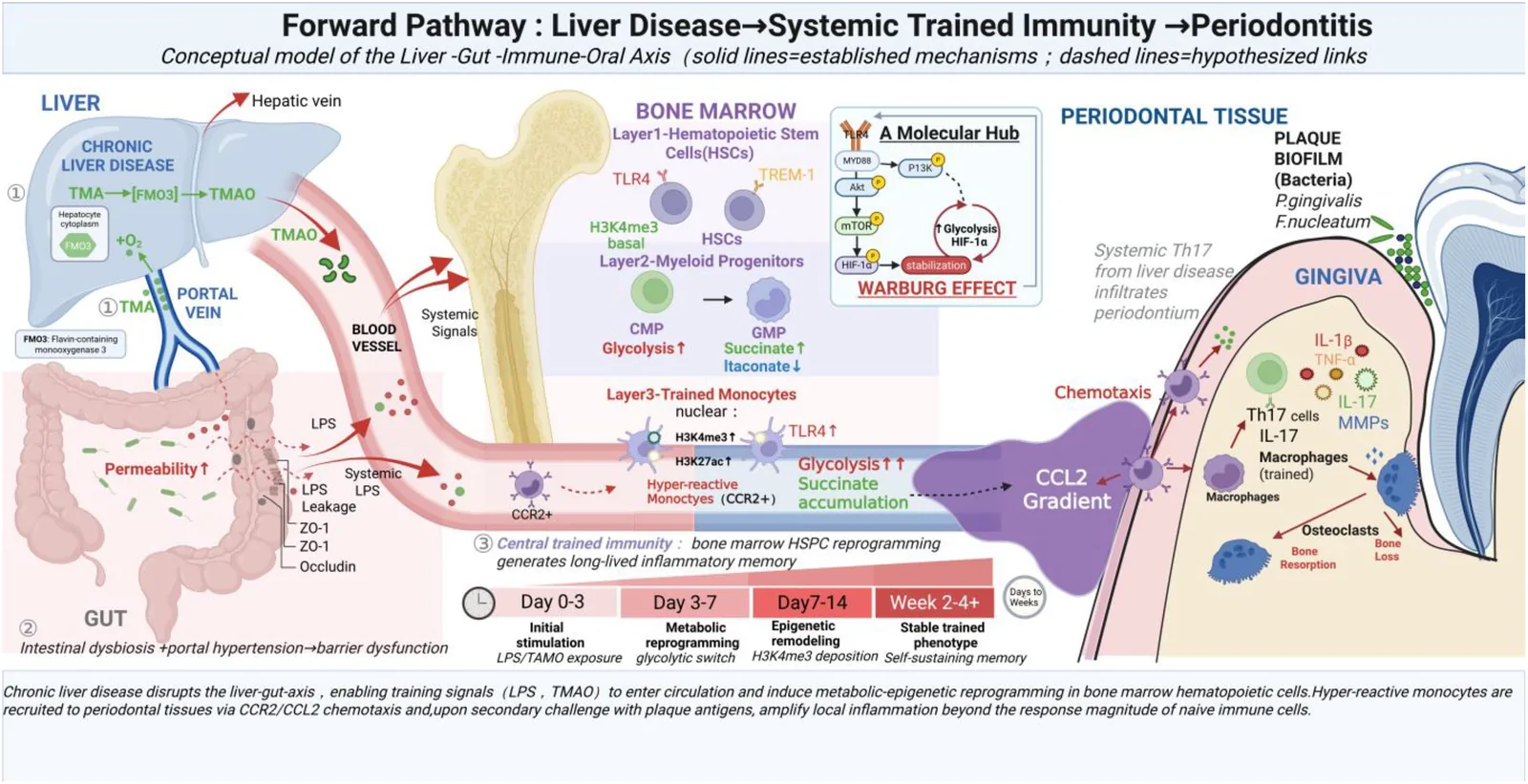
Conceptual model of the forward pathway in the liver-gut-immune-oral axis. Created in BioRender. Li, X. (2026) https://BioRender.com/acv33ws.

**Figure 2:**
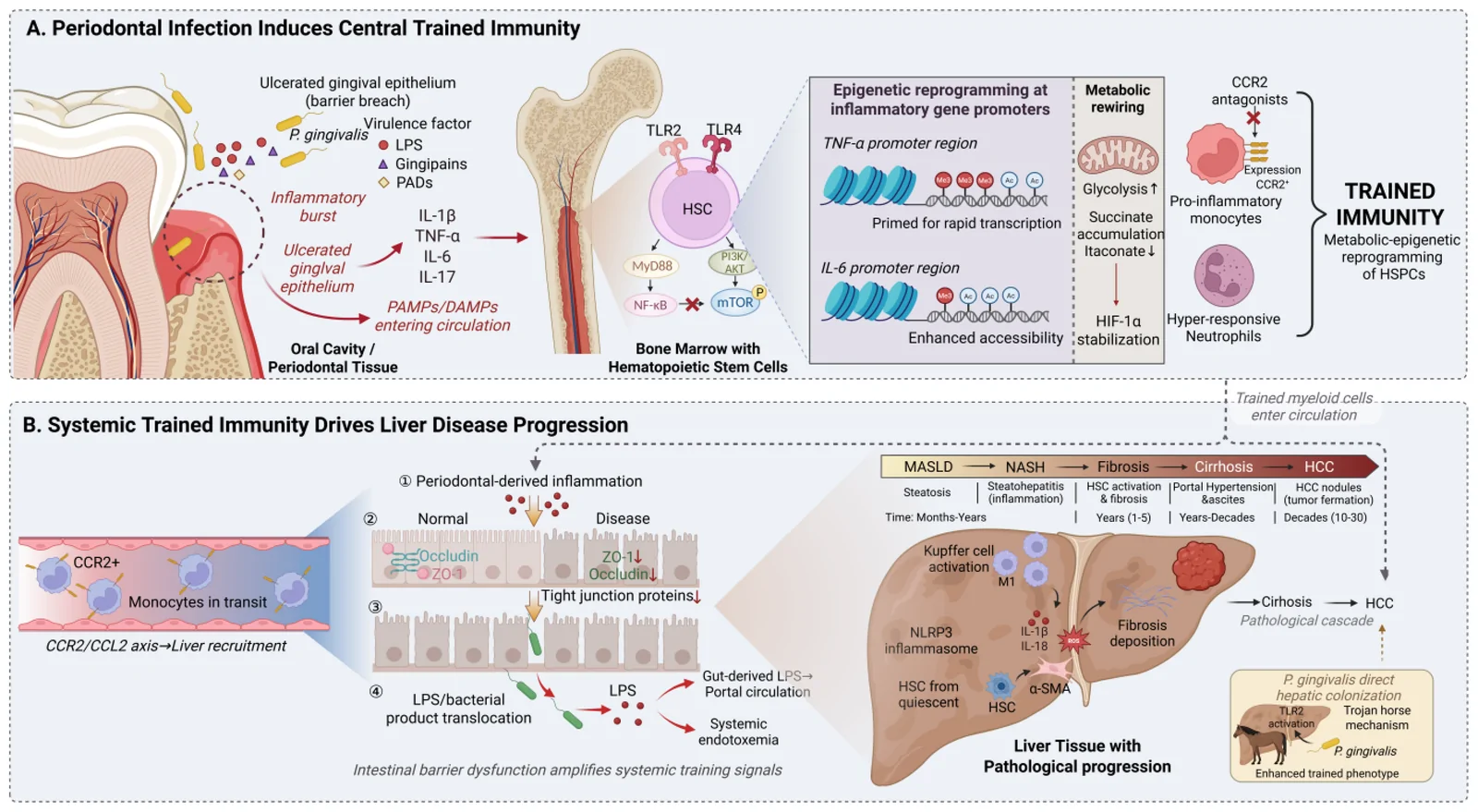
Conceptual model of the reverse pathway in the liver-gut-immune-oral axis. Created in BioRender. Li, X. (2026) https://BioRender.com/gz9290p.

The corrected **Figure 1**, and **Figure 2** appear below.

The original version of this article has been updated.

